# Are the Editors Responsible for Our Obsession with the Impact Factor?

**DOI:** 10.1128/mBio.02019-17

**Published:** 2017-12-19

**Authors:** Juan Carlos Argüelles, Raquel Argüelles-Prieto

**Affiliations:** a Área de Microbiología, Facultad de Biología, Universidad de Murcia, Murcia, Spain; b Laboratorio de Cronobiología, Facultad de Biología, Universidad de Murcia, Murcia, Spain

**Keywords:** editors, impact factor, scientific publication

## LETTER

The so-called impact factor (IF), introduced in an attempt to order scientific journals according to their influence in a particular field, is a dangerous instrument more related to competition among journals, which proudly wear their IF badges as a sign of status, than with scientific research (1). Moreover, the IF also constitutes an essential element in the daily activity of many researchers, who seek, sometimes obsessively, to publish their work in journals with a high impact. More dangerously still, the IF has become a tool of scientific policy, distorting scientific activity and its perception (1–4).

Although the reasons for this widely extended misuse of the IF are complex, the accountability of scientists themselves may be taken as a clear manifestation of the economic and sociopolitical theory known as the “tragedy of the commons,” in which the short-term self-interest of individuals prevails over the global profit of public good (“common”) (3). Therefore, instead of benefiting from personal contributions, the “common” becomes actually hampered. Applied to scientific communications, this policy seriously impairs the progress of science considered “common” because only restricted groups receive great credit, sometimes deservedly, by placing their articles in high-IF journals (2, 3). As a consequence, the rest of the scientific community is forced to follow this stratagem, altering the process of scientific creation, whose priority should be discovery rather than publication. In this context, an unforeseen and striking breakthrough occurred 1 year ago when the editors in chief of ASM journals adopted a collegiate decision to eliminate any reference to the IF in the corresponding journal websites (5). The repercussions of this brave attitude are unpredictable but deserve to be accompanied by careful reflection regarding the ASM editorial policy (6).

However, blaming scientists for adopting the IF as a criterion for submitting their work for publication is as easy as it is unfair. They cannot choose freely, since it is not only their own professional reputation that is at risk but frequently the very survival of their research groups. Today, the concession of research projects, grants, or fellowships is in the hands of panels, whose members are reduced in number and are not specialists in all of the fields under review. Although their final decision is supposedly based on rigorous reports issued by recognized experts, there is always a certain discretionary margin. The scientific community has universally accepted that the so-called “top journals” only publish the most relevant papers, which are at the frontier of new knowledge. Thus, the IF emerges as a “unifying parameter” that applies equally to all research groups, regardless of their line of work. As a consequence, two journals with the same IF but from different fields will have identical scientific value for the evaluators, who assume the degree of a journal’s relevance to be applicable to all published articles, with no exception, making it impossible to measure the true single value. Viewed through this prism, the IF might be considered an “objective leveling index,” although it would be dangerous to use any kind of statistical data out of context (1–3).

Quite apart from the above, we think it is important to analyze the responsibility of journals’ editors in the possible misuse of the IF. Indeed, and no doubt involuntarily, the editors themselves play a part in this obsession with the IF, leaving aside, of course, their unquestionable ethical behavior. During their tenure, they are the guardians of their journal’s prestige and it is only logical that they wish to publish the work of consolidated groups of renown. As a counterpart, they may be more demanding with groups of less acclaim in their field or those that propose novel hypotheses that are not immediately sustainable by hard experimental evidence.

Therefore, editors have become rigorous filters of quality control. It is common to receive a responsive message stating that, while our work is excellent, its publication exceeds the capacity of the journal. Thus, the corresponding editor regrets that it cannot be considered for review because so many good papers are received in the editorial office and, unfortunately, they are forced to reject most of them. On other occasions, the trouble is that the manuscript is “beyond the scope” of the journal in question. Strictly speaking, this is not the editor’s fault because, after all, he or she is responsible for the yearly IF review. However, it is perhaps questionable to what extent the prestige and scientific weight of a group (i.e., its IF) should be determining factors in the initial acceptance of an article for review while papers of a similar level from less well-established groups are rejected.

As a corollary, we are firmly convinced that editors should be prevented from submitting research articles to the journal that they serve. Beyond the fact that they act as filters of the articles’ scientific quality, editors must frequently resolve discrepancies between authors and reviewers and have the final decision on a paper’s acceptance. Therefore, submissions from their own research group will, almost inevitably, be judged with more permissive criteria. Because each area is served by a large number of journals, it would seem very easy to avoid such doubt: “Caesar’s wife must be above suspicion.”
